# Diffusion modeling reveals effects of multiple release sites and human activity on a recolonizing apex predator

**DOI:** 10.1186/s40462-021-00270-w

**Published:** 2021-06-30

**Authors:** Joseph M. Eisaguirre, Perry J. Williams, Xinyi Lu, Michelle L. Kissling, William S. Beatty, George G. Esslinger, Jamie N. Womble, Mevin B. Hooten

**Affiliations:** 1 Department of Natural Resources and Environmental Science, University of Nevada Reno, Reno, NV USA; 2 United States Fish & Wildlife Service, Marine Mammals Management, Anchorage, AK USA; 3 Department of Statistics, Colorado State University, Fort Collins, CO USA; 4 U.S. Geological Survey, Alaska Science Center, Anchorage, AK USA; 5 Southeast Alaska Inventory and Monitoring Network, National Park Service, Juneau, AK USA; 6 Glacier Bay Field Station, National Park Service, Juneau, AK USA; 7 Colorado Cooperative Fish and Wildlife Research Unit, U.S. Geological Survey, Department of Fish, Wildlife, and Conservation Biology, Colorado State University, Fort Collins, CO USA; 8 Present address: Wildlife Biology Program, Department of Ecosystem and Conservation Sciences, W.A. Franke College of Forestry and Conservation, University of Montana, Missoula, MT USA; 9 Present address: U.S. Geological Survey, Upper Midwest Environmental Sciences Center, La Crosse, WI USA

**Keywords:** Bayesian, Biological invasion, Ecological diffusion, Partial differential equation, Reaction-diffusion, Reintroduction, Sea otter

## Abstract

**Background:**

Reintroducing predators is a promising conservation tool to help remedy human-caused ecosystem changes. However, the growth and spread of a reintroduced population is a spatiotemporal process that is driven by a suite of factors, such as habitat change, human activity, and prey availability. Sea otters (*Enhydra lutris*) are apex predators of nearshore marine ecosystems that had declined nearly to extinction across much of their range by the early 20th century. In Southeast Alaska, which is comprised of a diverse matrix of nearshore habitat and managed areas, reintroduction of 413 individuals in the late 1960s initiated the growth and spread of a population that now exceeds 25,000.

**Methods:**

Periodic aerial surveys in the region provide a time series of spatially-explicit data to investigate factors influencing this successful and ongoing recovery. We integrated an ecological diffusion model that accounted for spatially-variable motility and density-dependent population growth, as well as multiple population epicenters, into a Bayesian hierarchical framework to help understand the factors influencing the success of this recovery.

**Results:**

Our results indicated that sea otters exhibited higher residence time as well as greater equilibrium abundance in Glacier Bay, a protected area, and in areas where there is limited or no commercial fishing. Asymptotic spread rates suggested sea otters colonized Southeast Alaska at rates of 1–8 km/yr with lower rates occurring in areas correlated with higher residence time, which primarily included areas near shore and closed to commercial fishing. Further, we found that the intrinsic growth rate of sea otters may be higher than previous estimates suggested.

**Conclusions:**

This study shows how predator recolonization can occur from multiple population epicenters. Additionally, our results suggest spatial heterogeneity in the physical environment as well as human activity and management can influence recolonization processes, both in terms of movement (or motility) and density dependence.

## Background

The global decline of apex predators has changed ecosystems [1–4]. These changes continue to have cascading effects across trophic levels, resulting in new ecosystem states of varying resilience [2]. When an apex predator is reintroduced, however, such a perturbation followed by continued growth and expansion of the population can change ecological communities and revert an ecosystem to a previous state [5]. Although often controversial, such shifts in ecosystem state can achieve conservation goals and afford ecological and economic benefits [6].

Predator reintroductions are sometimes proposed to recover ecosystem services or remedy human-caused declines, such as those due to overharvest [6, 7]. One of the most successful and celebrated efforts has been the reintroduction of wolves (*Canis lupus*) and subsequent recovery in the Greater Yellowstone Ecosystem. Wolves recolonized the area at a rate of about 10 km per year [8], and their renewed presence mediated over-browsing by elk (*Cervus canadensis*) and allowed the previous vegetation structure to return, subsequently driving additional recovery across the ecosystem [5]. Many reintroductions are unsuccessful, however [9, 10], because the distributions of resources, sources of mortality, and the physical environment—factors that influence recolonization—are highly variable through space and time [11–13]. Recolonization by apex predators is thus spatiotemporally dynamic, especially over large geographic areas that are characterized by finer-scale ecological variability [7]. Therefore, recolonization by a predator, as well as its abundance and persistence, will vary over space and through time.

Sea otters (*Enhydra lutris*), apex predators of nearshore marine systems, were harvested during the commercial fur trade up until the early 20th century, at which point they had declined nearly to extinction across most of their range [14]. For decades following, the nearshore marine ecosystems in many areas transitioned to, and persisted in, alternative states dominated by benthic herbivores that sea otters normally prey upon [15]. Legislation, beginning with the Fur Seal Treaty (1911), followed by the Marine Mammal Protection Act (MMPA; 1972) and the Endangered Species Act (1977), protected sea otters from harvest, with the exception of harvest by Alaska Natives for subsistence and handicraft, per the MMPA [14]. This protection facilitated sea otter population growth and expansion across parts of their range, which has been reverting the nearshore ecosystem in some of these areas to the historical predator-dominated state [6, 15–17].

**Fig. 1:**
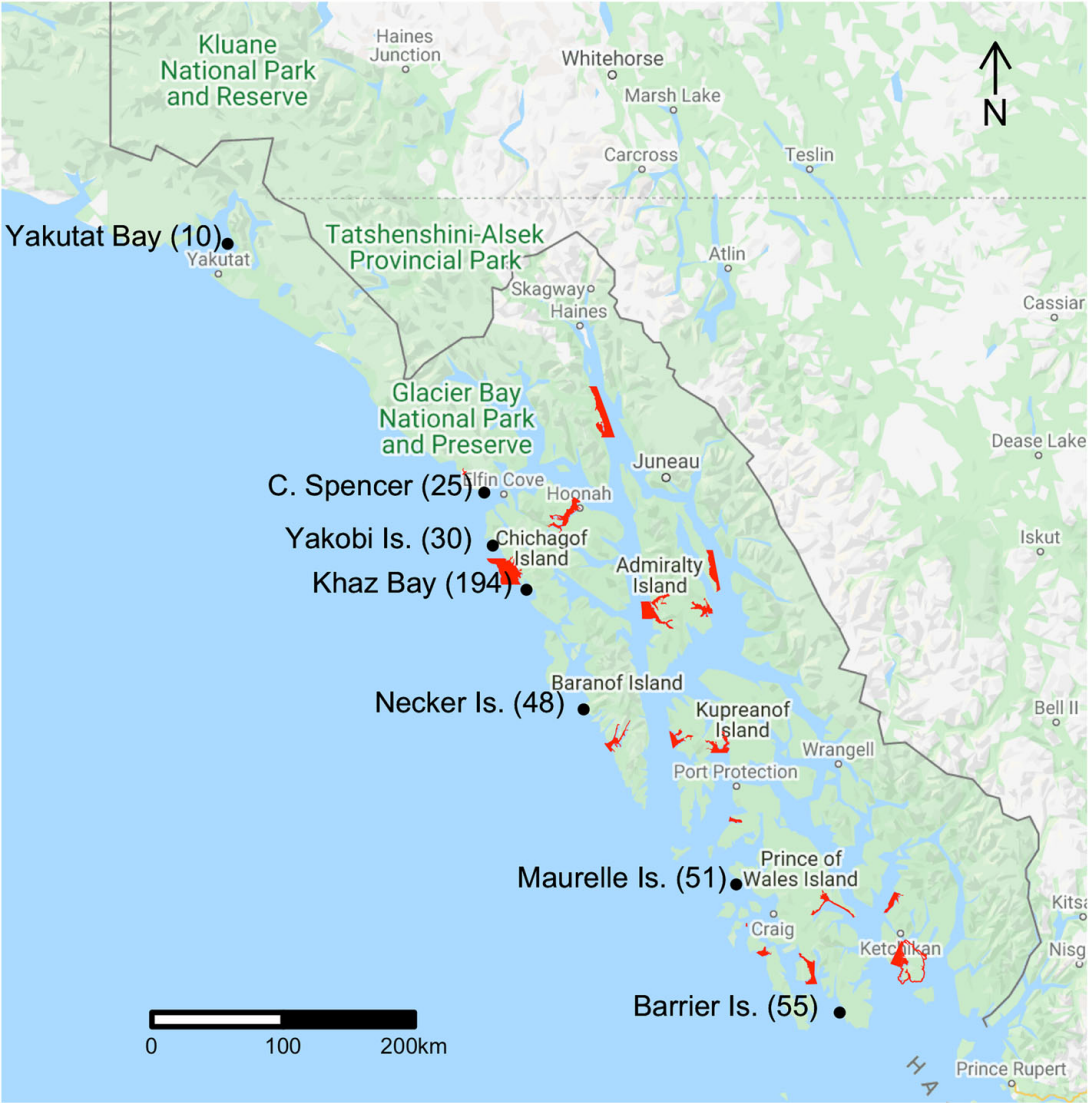
Map of Southeast Alaska showing the seven sites where sea otters were reintroduced 1965-69. Number of individuals released at each site given in parentheses. Red areas are closed to commercial fishing for prey species important to sea otters

One of these areas is Southeast Alaska, where during the late 1960s, the then grazer-dominated nearshore system was perturbed by the translocation of 413 otters from stable remnant populations in Prince William Sound and around Amchitka Island, Alaska [18]. This reintroduction followed previous failed attempts and was a four-year effort that translocated sea otters to seven sites across Southeast Alaska (Fig. 1). The number of individuals released at each site ranged from 10–194 [14, 18]. These individuals seeded a population that was recently estimated to exceed 25,000 [19].

The growing and expanding population colonized previously occupied areas, as well as newly available habitat that was historically glaciated (e.g., Glacier Bay; [20]). Across Southeast Alaska, the sea otter population is likely decades from reaching carrying capacity [19, 21]; even in Glacier Bay, the most densely populated area, evidence suggests carrying capacity may not be reached for 30 years [21].

Recent studies of the sea otter population in Southeast Alaska used integrated data models to investigate regional population trends and density-dependent effects [19] and influence of subsistence harvest [22]. While the approaches applied in these studies accounted for movements between discrete sub-regions within Southeast Alaska (i.e., immigration and emigration), they assumed a known intrinsic growth rate and did not explicitly incorporate a mechanistic model of population spread that would naturally capture movements of recolonizing individuals throughout this continuous geographic area. Spatiotemporal models, including those based on ecological diffusion, allow incorporating such dynamics and can provide novel insight beyond what conventional methods yield [23]. Therefore, several questions regarding the recolonization dynamics of this and other apex predators, as well as how they drive transitions from grazer- to predator-dominated ecosystem states, remain.

For example, while wolves and their resources were protected by a national park during the early stages of their recolonization of the Greater Yellowstone Region, sea otters in Southeast Alaska faced immediate competition with commercial fishing industries for some of their primary prey (e.g., urchins *Strongylocentrotus* spp. and bivalves *Panopea* spp.) as well as mortality from subsistence harvest [24, 25]. Indeed, sea otter population growth and spread was remarkable after individuals reached Glacier Bay National Park—the only area in Southeast Alaska where subsistence harvest of sea otters is not permitted—around the mid 1980s [20], yet Southeast Alaska encompasses a diverse matrix of marine areas with various types of resource management. This recolonization event affords the opportunity to assess how natural resource management can influence predator recolonization dynamics. Further, given the multi-site nature of the reintroduction, we also have an opportunity to investigate how population growth and spread can vary among population epicenters and how multi-site reintroductions may influence the success of recolonization.

We address some of the remaining questions about predator recolonization dynamics using a mechanistic spatiotemporal model of ecological diffusion that accounts for density dependent population growth and the spread of the population from multiple reintroduction sites. In particular, we examined the growth and spread of sea otters in Southeast Alaska to (1) investigate how colonizing individuals moved throughout the area from multiple reintroduction sites and (2) determine what factors contributed to the long term persistence of sea otters in particular locations, with a focus on the influence of managed areas (e.g., where limited or no commercial fisheries exist and/or where subsistence harvest of sea otters does not occur). Our approach involved integrating the mechanistic model of population growth and spread in a Bayesian hierarchical framework to estimate process parameters and uncertainty [26]. This approach has previously been applied on much smaller spatial scales to model sea otter recolonization and population dynamics in Glacier Bay from a single epicenter [20, 21, 27]. Here, we applied it across seven population epicenters (or reintroduction sites) to learn about changes in distribution and abundance of sea otters in a region with spatially-variable management regimes.

## Methods

### Data collection

Various aerial survey methods have been used to collect data on the distribution and abundance of sea otters in Southeast Alaska. These include design-based, distribution, and model-based aerial photographic surveys.

**Design-based surveys** Design-based aerial surveys [28, 29] were implemented in Yakutat Bay in 1995 and 2005, Glacier Bay in 1999–2004, 2006, and 2012 [30], and across the remainder of Southeast Alaska in 2002, 2003, 2010, and 2011 [31]. These surveys involved observers counting sea otters along 400-m wide linear transects flown with single-engine high-winged aircraft at a speed of 104 km/hr and altitude of 91 m. Transects were stratified based on depth and distance to shore, where areas with depths ≤40 m and closer to shore received greater sampling effort.

These design-based surveys also incorporated intensive search units (ISUs) to use in estimating detection probability; sea otters frequently dive beneath the surface to forage, during which time that are not available for detection [27]. During the survey, approximately every 15 minutes, an ISU was initiated based on the presence of a group of 1–20 sea otters. After being counted initially, the ISUs were re-counted while the pilot flew five concentric 400-m diameter circles so that a final count of each group could be obtained. In total, greater than 20,000 km of transects were flown across Southeast Alaska, and details of this effort were outlined recently by [19].

**Distribution surveys** We used data from distribution surveys only when design-based data were unavailable. This included Glacier Bay in 1993, 1996–1998, 2005, 2009, and 2010. Distribution surveys were conducted by fixed-wing aircraft with one or more observers and focused on favorable marine habitats (i.e., areas where depth was <40 m; [20]). The locations and counts of all groups of sea otters encountered were recorded by the observer(s).

**Aerial photographic surveys** Aerial photographic surveys [32] were conducted in Glacier Bay in 2017, 2018, and 2019 [33]. Aerial photographic surveys were conducted from a single-engine high-winged aircraft with a high-resolution DSLR camera (Nikon D810, 36.6 megapixel) with an 85 mm focal length lens (Zeiss F/1.4 ZF.2) mounted in a porthole in the belly of the aircraft. Random and optimized (see [34]) linear transects were flown at a speed of 157–166 km/hr and altitude of 213–250 m with the camera capturing overlapping images. Each image covered ∼60 m ×90 m area of the water’s surface. We used only non-overlapping images for analyses [21].

### Hierarchical model of ecological diffusion

We modeled the growth and spread of sea otters across Southeast Alaska using an ecological diffusion model. Ecological diffusion of a population through space and time emerges from the movements of many individuals following random walks with spatially heterogeneous movement probabilities [35]. Over time, individuals congregate in favorable areas, where they exhibit longer residence time, giving rise to spatiotemporal variability in population distribution and abundance.

#### Model specification

We modeled sea otter abundance in Southeast Alaska at locations $i=1,\dots, q$, where *q* is the total number of 400 ×400 m grid cells in the study area, during time $t=1970,\dots,2020$. Note that modeling on this 400 m spatial resolution matches the resolution of the design-based surveys. Due to the finer spatial resolution, the aerial photographic survey counts were aggregated to the 400 m scale, following [21]. Due to imperfect detection and availability of sea otters during surveys, we modeled the relationship between the latent true abundance of sea otters *N*_*i*,*t*_ and observed relative abundance *y*_*i*,*t*_ as

1 $$ y_{i,t} \sim \text{Binomial}(N_{i,t},p_{t}),  $$

where *p*_*t*_ is the detection probability, defined here as the probability that an animal is on the surface and available to be counted. ISU data were collected during 12 years of the design-based surveys, allowing estimation of detection probability. Additionally, we used a moment-matched prior for three years for which aerial photo surveys were conducted; the moments were matched to the marginal posterior of the detection probability estimated by [34] (see Appendix 1).

We modeled true abundance with a negative binomial distribution conditioned on a dynamic mean *λ*_*i*,*t*_ and dispersion parameter *τ*:

2 $$ N_{i,t} \sim \text{NB}(\lambda_{i,t},\tau).  $$

The intensity parameter, *λ*_*i*,*t*_ is the expected sea otter abundance in the *i*th grid cell during time *t*. Because diffusion is a continuous process, we obtain *λ*_*i*,*t*_ by integration over a location $\mathcal {S}_{i}$

3 $$ \lambda_{i,t}=\int_{\mathcal{S}_{i}} \lambda(\mathbf{s},t)d\mathbf{s},  $$

where *λ*(**s**,*t*) is the population intensity at any location **s**≡(*s*_1_,*s*_2_)^′^ in the continuous spatial domain.

We modeled the spatiotemporal dynamics to account for spread and density-dependent growth of the sea otter population with the following reaction-diffusion equation [21]:

4 $$  {}\frac{\partial}{\partial t} \lambda(\mathbf{s},t) \,=\, \left(\! \frac{\partial^{2}}{\partial s^{2}_{1}} \,+\, \frac{\partial^{2}}{\partial s^{2}_{2}}\!\right) \delta(\mathbf{s}) \lambda(\mathbf{s},t) + \gamma\lambda(\mathbf{s},t)\left(1 \,-\, \frac{\lambda(\mathbf{s},t)}{K(\mathbf{s})}\right).  $$

The diffusion coefficients *δ*(**s**) represent motility and are inversely proportional to residence time [35, 36]. The parameter *γ* is the intrinsic population growth rate, and *K*(**s**) accounts for density-dependence that may vary over space. While *δ*(**s**) controls how the population spreads, *K*(**s**) controls how many individuals areas can sustain long term. Note that *K*(**s**) corresponds to local density dependence, and the nominal carrying capacity of the region can be obtained by $\int _{\mathcal {S}} K(\mathbf {s}) d\mathbf {s}$ [21].

Equation (4) requires specifying an initial condition for *λ*(**s**,*t*_0_). A scaled Gaussian kernel can represent a single epicenter from which a population spreads [37]. However, given that sea otters were reintroduced at seven sites throughout Southeast Alaska, we used a sum of *J*=7 scaled Gaussian kernels, each centered on a reintroduction site (or epicenter) **d**_*j*_:

5 $$ \lambda(\mathbf{s},t=1970)=\sum_{j=1}^{J} \frac{\theta_{j} \text{exp}\left({\frac{-||\mathbf{s}-\mathbf{d}_{j}||^{2}}{\kappa_{j}^{2}}}\right)}{{ \int_{\mathcal{S}}} \text{exp}\left(\frac{-||\mathbf{s}-\mathbf{d}_{j}||^{2}}{\kappa_{j}^{2}}\right) d\mathbf{s}}.   $$

*θ*_*j*_ is a scale parameter controlling the initial density of individuals at **d**_*j*_, and *κ*_*j*_ is a dispersion parameter controlling the initial isotropic spread of those individuals around **d**_*j*_. To limit population spread based on sea otter biology, we used a reflective boundary, which does not allow population spread past the boundary, at locations adjacent to terrestrial environments as well as at locations at the offshore edge of the nearshore system, i.e., locations exposed to open ocean that are 5 km from shore or exhibit depths >100 m, based on the distribution of survey observations.

To complete the specification of the hierarchical model, priors were specified for all model parameters. We used a combination of informative and weakly informative priors, based on previous results (e.g., from [20], [21], and [19]) as well as records of the translocations and historical observations [18]. We provide a complete list of priors in Appendix 1.

#### Environmental covariates

We expected that, over time, sea otters would congregate in areas with favorable habitat and resources. Thus, we modeled motility *δ*(**s**) as a log-linear function of covariates that have been found to be important drivers of sea otter space use and behavior [20, 21]. Based on previous studies, our covariates included depth, as a binary indicator (depth =1 where <40 m, and 0 otherwise), distance to shore, slope of the ocean floor, and shoreline complexity [20, 38–41]. Shoreline complexity was calculated for each location by summing the number of locations within a 1,000 m neighborhood that contained shoreline [20]. Given that subsistence harvest of sea otters [22] and human activities (e.g., disturbance from vessel traffic; [24]) influence sea otter population dynamics, we added a covariate of cumulative distance to the nearest incorporated city, town, or village. This was the sum of the shortest swimmable paths from each city, town, or village, to any location **s**.

As one of our goals was to investigate the varying levels of resource management across Southeast Alaska on the recolonization, we included Glacier Bay and fisheries closures as two indicator covariates, representing management categories. Sea otter population growth and recolonization dynamics are unique in Glacier Bay [19–21], which lies within a national park where various human activities (e.g., commercial fishing, subsistence harvest of sea otters, etc.) are limited. Some commercial fishing still occurs in Glacier Bay (i.e., for some finfish and Tanner crab *Chionoecetes bairdi*), but it is limited and being phased out. Red sea urchins (*Strongylocentrotus franciscanus*), sea cucumbers (*Parastichopus californicus*), and geoduck clams (*Panopea generosa*) are important prey for sea otters in Southeast Alaska [42–44], but they also support lucrative commercial fisheries [45]. Management of these state fisheries in Southeast Alaska involves a rotation of open and closed areas, in addition to areas that have remained closed long term due to federal jurisdiction, research, or being deemed not viable to support commercial harvest [45]; these areas closed long-term by regulation comprised what we termed ‘fisheries closures’ (Fig. 1). Dungeness crab (*Cancer magister*) are also important prey that are commercially harvested [46]; however, spatial data for this fishery were not available (but, we note that many of the Dungeness crab closures overlapped closures that we included). The log-linear function for motility was therefore

6 $$ {}\begin{aligned} \text{log}(\delta(\mathbf{s}))& = \beta_{0} + \beta_{1} \text{depth}(\mathbf{s}) + \beta_{2} \text{dist}(\mathbf{s}) + \beta_{3}(\text{slope}(\mathbf{s})\\ &\quad\times \text{depth}(\mathbf{s})) + \beta_{4} \text{shore}(\mathbf{s}) + \beta_{5} \text{town}(\mathbf{s})\\ &\quad + \beta_{6}\text{glba}(\mathbf{s}) + \beta_{7}\text{fish}(\mathbf{s}). \end{aligned}  $$

While modeling *δ*(**s**) as a function of covariates allows for investigating how the population spreads to reach certain areas, modeling local density dependence *K*(**s**) allows us to see if certain areas may influence long term population dynamics and densities. So, to further allow the process model to have sufficient flexibility to capture the unique colonization dynamics of Glacier Bay and to investigate the effects of resource management, including fisheries closures, on sea otter population dynamics within the ecological diffusion framework, we allowed density dependence to vary over space as a function of covariates. This took the form:

7 $$ \text{log}(K(\mathbf{s}))=\alpha_{0}+\alpha_{1} \text{glba}(\mathbf{s}) + \alpha_{2} \text{fish}(\mathbf{s}).  $$

While this formulation implies local density dependence (or local nominal carrying capacity) varies over the region only according to these two indicator covariates, realized carrying capacity depends on motility and thus the covariates driving it as well [21].

All covariates, except for the binary indicators, were centered and scaled to mean zero and unit variance for estimation.

#### Estimation, derived parameters, and model validation

We sampled from the posterior distribution of the hierarchical model with Markov chain Monte Carlo (MCMC), implemented in R and C++ [47]. Ecological diffusion (Eq. 4) is continuous in space and time, so we used finite differencing for estimation over the discretized spatial and temporal domains [21, 27, 36]. Due to the resolution of the data, we set the spatial discretization to 400 m ×400 m and the temporal discretization to *Δt*=1 d. Additionally, we used homogenization for computational feasibility [21, 27, 36, 48], which was described in detail by [21] for the logistic ecological diffusion model. We followed [27] and [21] and chose *ε*=1/10, which corresponds to a homogenized scale of 4 km ×4 km. Much of the computational demand of this and similar spatiotemporal models results from the high dimensional matrix operations required by the finite differencing procedure [27]. In contrast to previous work, we handled those as sparse matrix operations, which reduced the computational burden markedly.

To help understand how the colonization front of otters moved through space and time, we estimated the asymptotic spatially explicit spread (or colonization) rates. The asymptotic spread rate for the Malthusian (or exponential growth) model and the minimum spread rate for the logistic model is given by $2\sqrt {\bar {\delta }{\gamma }}$, where $\bar {\delta }$ is the homogenized diffusion coefficient [20, 49]. Asymptotic spread rates greater than the minimum are allowed in nonlinear (e.g., logistic) cases, and computing them requires knowing the shape of the wave front. From Eq. (5), the steepness of the front at *t*=1970 is $1/\kappa _{j}^{2}$, and from theory of propagating waves, we know that the shape of the wave front is conserved [50]. Finally, if the front is steep, i.e., $1/\kappa _{j}^{2}>\sqrt {\gamma /\bar {\delta }}$, then the spread rate converges to $2\sqrt {\bar {\delta }{\gamma }}$, whereas if the front is flat, i.e., $1/\kappa _{j}^{2}<\sqrt {\gamma /\bar {\delta }}$, asymptotic spread rate can be computed as $\frac {\bar {\delta }}{\kappa ^{2}_{j}}+\gamma \kappa ^{2}_{j}$ for any time *t*>1970 [50, 51].

We estimated total abundance $N(t)=\int _{\mathcal {S}} N(\mathbf {s},t) d\mathbf {s}$ by

8 $$ N^{(k)}(t)=\sum_{i=1}^{n_{0,t}} N_{i,t} + \sum_{m=1}^{n_{t}-n_{0,t}}\hat{N}_{m,t}^{(k)}+ \sum_{l=1}^{q-n_{t}}\Tilde{N}_{l,t}^{(k)}  $$

for the *k*th MCMC iteration. The term *N*_*i*,*t*_ is an observation of true abundance, $\hat {N}_{m,t}^{(k)}$ is posterior draw of true abundance where relative abundance was observed, $\Tilde {N}_{l,t}^{(k)} \sim \text {NB}(\lambda _{l,t}^{(k)},\tau ^{(k)})$ where no data were collected, *n*_*t*_ is the number of locations where relative abundance or true abundance was observed, and *n*_0,*t*_ is the number of locations where only true abundance was observed [21].

We used the posterior predictive distribution to assess model fit. A posterior predictive draw for an observation *y*_*i*,*t*_ is given by $\Tilde {y}^{(k)}_{i,t} \sim \text {Binomial}(\Tilde {N}^{(k)}_{i,t},p^{(k)}_{t})$. We compared these samples to the data point-wise by comparing the observed counts to the 95% credible intervals of the posterior predictive counts [52]. We assessed convergence to the posterior by visual inspection of the MCMC chains with traceplots. We summarized our parameter estimates using posterior means and 90% credible intervals [53, 54].

## Results

It required approximately seven days using 15 independent chains run in parallel to obtain an MCMC sample of 15,000 iterations from the posterior. Only 23 of 42,553 observed counts fell outside of the 95% posterior predictive intervals, suggesting no lack of fit over the area that was surveyed.

**Fig. 2:**
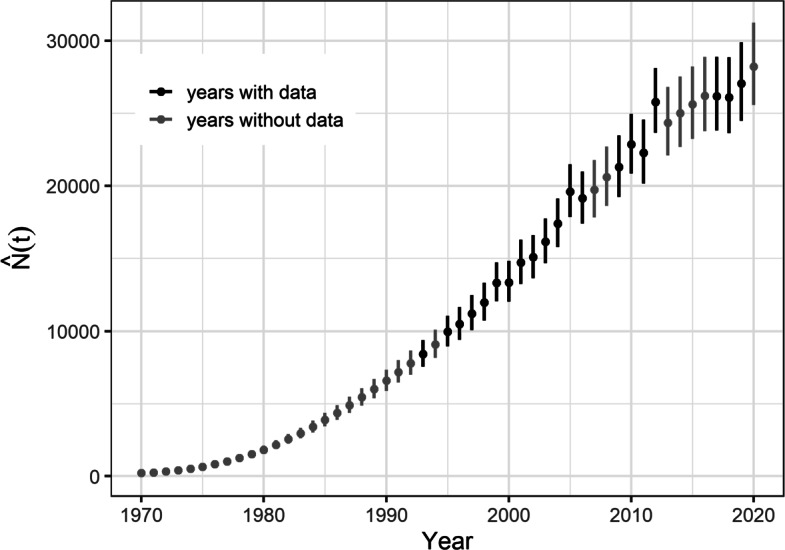
Time series of total abundance estimates from the spatiotemporal model of sea otter population growth and spread in Southeast Alaska. Points are posterior means, and segments are 95% credible intervals. Note region-wide surveys were completed over two years for the years 2002–2003 and 2010–2011

We estimated an intrinsic growth rate of about 0.29 (0.28, 0.31; Table 1). Our estimates of total abundance (Fig. 2) were similar to other recent estimates [19] and those obtained with the design-based estimator [55]. Although not definitive, it appears the consistently high annual growth rate of the sea otter population across Southeast Alaska may have begun to slow in the last few years (Fig. 2).

**Table 1:** Posterior means and 90% credible intervals for the parameters of the ecological diffusion model with logistic growth estimated for the sea otter population in Southeast Alaska 1970 to 2020. The subscripts on *θ* and *κ* are abbreviations of the translocation sites shown in Fig. 1. Estimates of detection probabilities are provided in Appendix 2: Table 2

Parameter	Lower bound	Mean	Upper bound
*β*_0_ (intercept)	16.25	16.36	16.48
*β*_1_ (depth)	-1.89	-1.77	-1.66
*β*_2_ (distance to shore)	0.21	0.29	0.35
*β*_3_ (slope × depth)	0.14	0.22	0.29
*β*_4_ (shoreline complexity)	0.14	0.17	0.21
*β*_5_ (distance to towns)	0.37	0.45	0.55
*β*_6_ (Glacier Bay)	-0.37	-0.24	-0.10
*β*_7_ (fisheries closures)	-1.48	-1.34	-1.16
*α*_0_ (intercept)	-1.77	-1.66	-1.55
*α*_1_ (Glacier Bay)	2.78	3.16	3.58
*α*_2_ (fisheries closures)	0.12	1.87	6.91
*γ* (intrinsic growth)	0.28	0.29	0.31
*τ* (overdispersion)	0.03	0.03	0.03
*θ*_MI_ (initial density)	119.65	147.53	175.46
*θ*_BI_	8.06	9.75	11.40
*θ*_NI_	8.33	9.96	11.63
*θ*_KB_	65.41	98.90	132.59
*θ*_YB_	8.36	10.01	11.69
*θ*_YI_	63.74	96.18	128.84
*θ*_CS_	68.19	98.82	130.55
*κ*_MI_ (initial dispersal)	25.41	28.78	32.20
*κ*_BI_	1.37	2.54	3.80
*κ*_NI_	4.42	9.11	13.90
*κ*_KB_	0.51	0.63	0.78
*κ*_YB_	0.80	2.23	3.79
*κ*_YI_	4.17	8.49	12.40
*κ*_CS_	4.59	9.23	13.91

We also found evidence that all covariates included in the model had an effect on the spatiotemporal process, both in terms of motility and density dependence (Table 1). Generally, sea otters across Southeast Alaska seemed to prefer areas with shallow depth (i.e., <40 m), close to shore, steeper slopes (in areas with shallow depth), and straighter shorelines (Table 1). Additionally, sea otters tended to concentrate in Glacier Bay, areas with fisheries closures, and areas close to human communities, although the effect size was relatively large for areas with fisheries closures compared to Glacier Bay and human communities (Table 1). Further, population densities that begin to regulate growth were likely highest in Glacier Bay, followed by areas with fisheries closures, and lowest elsewhere in the region, although there was overlap in credible intervals between the effects of the protected status of Glacier Bay and areas with fisheries closures (Table 1).

**Fig. 3:**
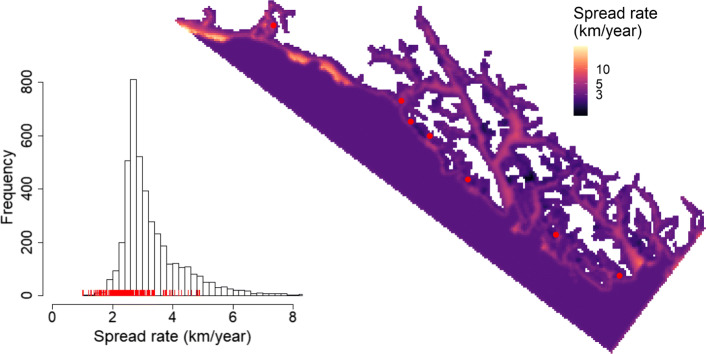
Asymptotic spread rates of the sea otter population in Southeast Alaska based on parameters estimated in the ecological diffusion model. Note that the map is presented on the homogenized (4 km) resolution, and the red points represent the epicenters (translocation sites). On the left is a histogram showing the values presented in the map on the right. Note the *x*-axis is truncated for presentation. Red vertical lines represent the spread rates in areas closed to commercial fishing for prey species important to sea otters

The initial dispersal conditions suggested a steep wave front (i.e., satisfied $1/\kappa _{j}^{2}>\sqrt {\gamma /\bar {\delta }}$), so we estimated asymptotic spread rates with $2\sqrt {\bar {\delta }{\gamma }}$ across all epicenters. Rates varied primarily from about 1–8 km/yr, with a median of 3.0 km/yr, but areas further from shore commonly exhibited more rapid spread rates (Fig. 3). Additionally, areas with fisheries closures generally exhibited slower spread rates (Fig. 3).

## Discussion

**Fig. 4:**
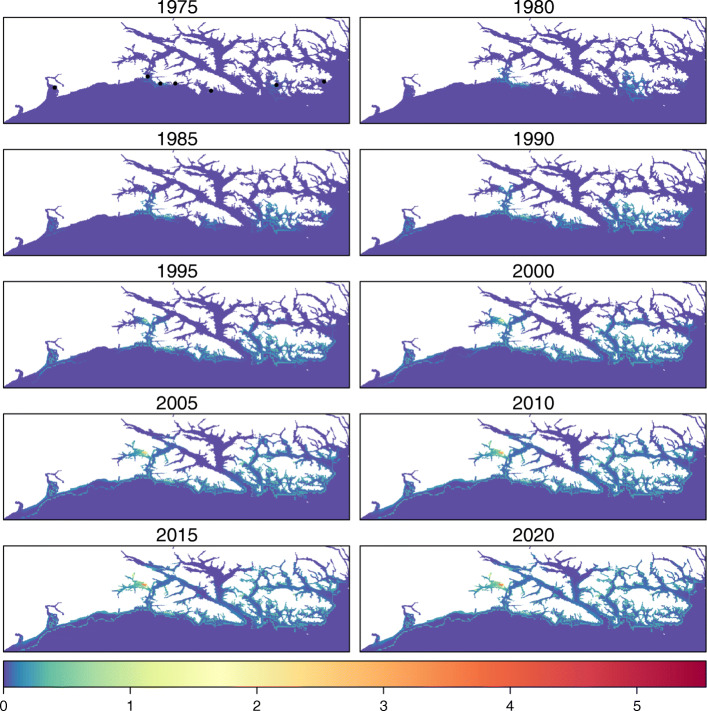
Expected abundance (*λ*_*i*,*t*_) of sea otters in Southeast Alaska estimated with the ecological diffusion model. Note the study area was rotated counterclockwise for presentation. Black points in the first panel correspond to the epicenters (translocation sites)

To improve our understanding of the reintroduction biology of apex predators [13], we modeled the ongoing recolonization of Southeast Alaska by sea otters as a spatiotemporal process based on ecological diffusion, accounting for multiple population epicenters (i.e., reintroduction sites), preferential dispersion, and spatially-variable density dependence (Fig. 4). In addition to the novelty of spatially-varying density dependence, to our knowledge, this is the largest spatial extent and finest spatial reso- lution over which such a model has been implemented. Homogenization offers substantial computational gains [36, 48], but we also used sparse matrix operations, which made implementing the model on the scale of Southeast Alaska much more computationally tractable.

Ecological diffusion is well established in mathematical and ecological theory pertaining to the spread of organisms [35]; however, other process models could certainly be used to model recolonizing populations. For example, [56] implemented a dynamic occupancy model where colonization proceeds following gradients of favorable habitat. While an extension to modeling abundance in their framework is certainly possible, ecological diffusion naturally models abundance as well as movement toward and concentration in favorable habitat. [19] and [38] modeled sea otter recolonization (of Southeast Alaska and the central coast of California, respectively) by parsing the study areas into distinct units and specifying immigration and emigration among them. While doing so offers computational advantages, inferences are restricted to those defined units. In contrast, with a continuous spatiotemporal model, such as ecological diffusion, inferences can be made about any areas of interest within the modeled domain—defined a priori or *a posteriori*—based on straightforward *post hoc* calculations (e.g., time series of abundance within different areas).

Our results from applying the ecological diffusion model to Southeast Alaska indicate that sea otters generally concentrate in areas presumed to be favorable for foraging as well as areas closer to human communities, but sea otter densities that begin to regulate population growth are higher in areas with limited or no commercial fishing and other human activities. We were also able to obtain greater precision in our estimates of total abundance than design-based estimators and recent modeling efforts (Fig. 2; [19]). Furthermore, we found that the rates of colonization averaged about 3 km/yr throughout the region, with higher rates being in areas with higher motility. These factors all contributed to the ongoing success of the recolonization that continues to drive ecosystem change in the region [15, 42].

### Spatial variability in abundance

Another effort to model the growth and expansion of the sea otter population in Southeast Alaska found that abundance and carrying capacity varied between large, discrete sub-regions [19]. However, we also accounted for how spatial covariates drive variation in abundance and movement of sea otters throughout the region (Figs. 3 & 4). We found that shallow depth, which defines foraging habitat [39, 40], was positively correlated with sea otter residence time. We also found that sea otters additionally concentrate in areas where commercial fisheries are closed, as well as in protected areas, where subsistence harvest of sea otters is not permitted (i.e., Glacier Bay; Table 1), which suggests greater prey availability, foraging habitat, and mortality risk are strong drivers of sea otter distribution and abundance. Our finding that sea otters exhibit higher residence time closer to human communities is seemingly inconsistent with previous findings of exposure to subsistence hunting influencing sea otter movement [44] and population growth [22]. However, the diffusion model is likely capturing what happens during the initial colonization of areas closer to communities, and, in the longer-term, areas closer to communities where harvest is common may act as population sinks [22].

Sea otter colonization and foraging habits have marked effects on assemblages of benthic invertebrates, including many commercially-harvested shellfish [42, 44, 57]. While some areas are closed to commercial fishing in Southeast Alaska due to seemingly unsupportable abundances of harvested species (for commercial purposes), sea otters are adept at capturing these species even in closed areas. [42] found that sea cucumber abundance negatively correlates with sea otter occupancy but also observed sea cucumbers in sea otter diets in areas where surveys suggested a 100% decline in sea cucumbers. This finding suggests that sea otters can find prey even when it is undetected by targeted surveys. So, even in areas that may not be able to support commercial harvest by humans, an abundance of prey may be available to sea otters, including sea cucumbers but likely other species as well that may have become abundant as the ecosystem transitioned to the predator-dominated state [58].

In addition to influencing residence time of sea otters, these areas with reduced commercial activity may offer sea otters some relief from competition for food resources, thereby supporting the higher nominal carrying capacities suggested by our results (Table 1). Furthermore, extensive glacial retreat over the last 350 years and subsequent ecological succession in the marine environment has led to a highly diverse and abundant benthic prey community in Glacier Bay since sea otters previously inhabited the region [20, 57]. In addition to the reduced human activity in the protected area of Glacier Bay, the new habitat likely further contributed to the higher nominal carrying capacity there (Table 1; [19, 21]). As the population expands into other previously-glaciated fjords with shallow habitat, we might expect such areas to similarly support higher carrying capacities, and we may be presented with an opportunity to investigate how novel niche space might interact with management strategy to drive spatially-variable carrying capacities.

In contrast to the relatively rapid recolonization of Southeast Alaska, sea otter populations have been slower to recover in the southern parts of their range, such as the coastal habitat of California. Parts of Southeast Alaska, such as the outer coast, are dominated by rocky benthos that can support healthy kelp forests. So, the top-down effects on grazers by sea otters would quickly release kelp from control [16], in turn providing otters with protective habitat. The California coast, on the other hand, is a matrix of disjunct rocky benthos and stretches of softer substrate—poor for persistent kelp forest establishment—that may limit the recovery of sea otter populations via limiting female dispersal and survival [59, 60]. Further, much more of the nearshore marine environment in California is exposed to open ocean compared to the more sheltered bays and passages of Southeast Alaska. Particularly exposed areas of the California coast (e.g., Point Conception) are thought to be barriers to sea otter population spread [60], and new evidence suggests sea otters may have utilized protected estuaries historically [61]. Predation by white sharks (*Carcharodon carcharias*) may also limit sea otter range expansion along the California coast [62]. These regional differences in recolonization dynamics highlight the need to carefully consider strategies to improve the likelihood of long-term success of predator reintroduction efforts.

Other studies have included multiple fine scale habitat covariates in population models to explain carrying capacity of sea otters [38]. As more spatial data become available for Southeast Alaska, similar covariates could be included in the diffusion model. However, homogenization of the logistic diffusion model implies that realized carrying capacity is, in part, a function of motility [21]. We included two indicator covariates in our formulation of *K*(**s**), representing areas with different management regimes, but variation in motility over the region also explains spatial variation in carrying capacity in the model. Other covariates, such as kelp canopy cover and benthic substrate composition, which have been shown to be important drivers of sea otter carrying capacity elsewhere [38], could be included in future models (i.e., as covariates affecting density dependence and/or motility).

### Colonization rates and multi-site reintroductions

While we estimated a median spread rate of 3.0 km/yr in Southeast Alaska, we also found that asymptotic spread rates of sea otters can vary greatly over such a vast region (Fig. 3). Asymptotic spread rates of recolonizing sea otters in California were first estimated to range from about 1.7 to 3.5 km/yr [63], and [60] estimated about 4.7 km/yr for the southern edge of the California range and about 2 km/yr for the northern edge. Similarly, [20] estimated rates of 1.5 to 4.5 km/yr in Glacier Bay. Recalling that these spread rates are estimated as $2\sqrt {\bar {\delta }{\gamma }}$ [20, 49, 63], the greater range that we estimated is due to (1) a higher intrinsic growth rate (discussed below; Table 1), as well as (2) the extensive spatial variability in motility harbored by a region of such size.

While we did not find evidence that any of the initial dispersal conditions (i.e., *κ*_*j*_) for sea otters in Southeast Alaska affected the theoretical asymptotic spread rates (Table 1), and thus the spatial variation of those rates did not vary among epicenters (Fig. 3), specific translocation strategies could improve colonization rates. For example, if individuals were released at a site such that they were spread out sufficiently to create a flat propagating front (i.e., satisfying the condition $1/\kappa _{j}^{2}<\sqrt {\gamma /\bar {\delta }}$), theory suggests the population could spread at rates greater than the minimum spread rate (i.e., $\frac {\bar \delta }{\kappa ^{2}_{j}}+ \gamma \kappa ^{2}_{j}$; [50, 51]). Somewhat counter-intuitively, this suggests that higher colonization rates could be achieved by reintroducing individuals over wide areas where the species is expected to have higher motility and thus lower residence time (i.e., less favorable habitat). While it is important to note that failed reintroductions are commonly attributed to translocations of low initial densities (resulting in elevated effects of demographic stochasticity and/or Allee effects) and to unsuitable habitat (causing high mortality; [13]), individuals released in areas correlating with low residence time should spread rapidly to several locations with more favorable habitat and begin to concentrate in those areas. In fact, our results provided evidence of this: The sea otter population spread quickly over areas with high motility, then settled at high abundance in areas with low motility, which included areas with limited or no commercial fishing (Figs. 3 & 4). Given the relationship between motility in the ecological diffusion model, population spread rates, and specific forms of resource selection functions [64], it is possible that preliminary investigations of individual animal movement—either in the reintroduction area or a similar area—could be used to optimize a reintroduction strategy in terms of the initial locations and densities of released individuals. Nonetheless, these inferences regarding improved translocation strategies are largely based on mathematical theory underlying diffusion models, so further study is needed to determine how they may apply to translocation and reintroduction efforts in practice.

While sea otter reintroductions along the North American coast were an early example of a multi-site effort [18], there is a recent and ongoing multi-site reintroduction of a terrestrial predator, fisher (*Pekania pennanti*), in the northwestern U.S. [65]. Similar to sea otters, fishers declined due to over-harvest and lack of management, yet reintroduction attempts have been showing promise in restoring this predator across its historical range [10]. The simulation modeling by [10] suggested that multiple reintroduction sites can improve the success of predator recolonization. Our work adds to this body of knowledge by documenting, with a mechanistic model fit to data, how such a process occurs over a region where colonizing individuals face variability in motility and density dependence. Indeed, our application was to a marine system, although a parallel application to the expanding fisher populations or similar terrestrial predator could reveal how such a process might vary between marine and terrestrial systems, over which animals have inherent differences in motility. Nonetheless, we found the spread rates of sea otters in Southeast Alaska were generally less than the 9.78 km/yr documented for wolves—highly mobile terrestrial predators [8]. Although, in certain areas, sea otter populations may be able to exceed that rate (Fig. 3).

### Intrinsic growth

A maximum growth rate of about 20–25% has been generally accepted for sea otter populations for some time [19, 66]. However, modeling the growth and spread of sea otters across the entire region of Southeast Alaska as a continuous spatiotemporal process suggested intrinsic growth for at least this population is higher (Table 1). The evidence was quite strong: We used an informative prior for *γ* centered on 0.25, based on previous studies, yet the data easily pulled the marginal posterior upward (Table 1; Appendix 1).

While the previous estimates were generally accepted, it had been suggested they were likely biased low due to underestimated natality [67]. Assuming female sea otters in the area have the ability to average about one female pup every other year, our estimated intrinsic growth of about 0.29 is reasonable and aligns with the requisite theoretical maximum population growth rate [66, 68]. It is therefore possible that sea otter populations have the potential to grow more rapidly when unhindered by density-dependent factors than previous evidence suggested. Indeed, our estimate of intrinsic growth is high among marine mammals [66, 69, 70] but is reasonable, especially because the relatively mild winter conditions and productivity of Southeast Alaska are likely conducive to sea otters averaging one pup per year.

Application of a diffusion model similar to the one we implemented revealed the intrinsic growth rate of wolves colonizing parts of France varied between about 0.3 and 0.7, depending on the amount of forest cover [71]. However, modeling intrinsic growth—the theoretical maximum rate of increase of the population—as a function of covariates, as [71] did, implicitly assumes that those covariates have a density-independent effect on population growth. In contrast, we chose to model the density dependence parameter *K*(**s**) as a function of spatial covariates because we hypothesized those covariates would affect how density moderates population growth (e.g., through reduced prey availability at higher population densities), rather than be density-independent.

### Continued population growth and spread

While it appears the annual rate of increase of the sea otter population in Southeast Alaska may be slowing (Fig. 2), it is likely still decades from reaching total carrying capacity [19, 21]. As the recolonization process continues, the population will reach new habitat, in addition to Glacier Bay, that will similarly afford greater local equilibrium abundances. Sea otters in the region also face growing conflicts with human interests and activities due to their effects on commercially-valuable and subsistence species [25]. However, the return of the historical state of the nearshore marine ecosystem is gaining support among many stakeholders because there is great value in the ecosystem services that the predator-dominated system can render, such as improved carbon sequestration, nursery habitat for fish, and greater fish biomass [6, 72].

As we continue to monitor this growing and expanding population, as well as the requisite ecosystem change, we can adapt our modeling approach to gain additional insight into total equilibrium abundance, the spatial variability of equilibrium abundance, the effects of subsistence harvest of sea otters and commercial fisheries, and how climate change may continue to influence the process. Key to this ongoing effort will be using the mechanistic diffusion model to forecast population growth and spread to dynamically optimize the monitoring framework (*sensu* [34]).

## Conclusions

As predator reintroductions continue to be proposed (e.g., 2020 Colorado Proposition 114), there is an increasing need to understand recolonization processes across modern land- and seascapes with varying levels of management and human activity. Fundamental to our understanding of how keystone predator reintroductions can drive ecosystem change is understanding how a predator population grows and expands its range. We provide new insight into how colonization and growth can occur from multiple reintroduction sites and with spatial heterogeneity in both the physical environment as well as human activity and management.

### Appendix 1: Priors

*γ*∼Normal(0.25,0.01^2^)

***β***∼Normal(**0**,10^2^**I**)

$\theta _{j} \sim \text {Normal}^{+}\left (\mu _{\theta,j},\sigma ^{2}_{\theta,j}\right) $, where ***μ***_*θ*_=(100,10,10,100,10,100,100)^′^ and $\boldsymbol {\sigma }^{2}_{\theta }=(20^{2},1^{2},1^{2},20^{2},1^{2},20^{2},20^{2})'$

$\kappa _{j} \sim \text {Normal}^{+}(\mu _{\kappa,j},\sigma ^{2}_{\kappa,j}) $, where ***μ***_*κ*_=(10,2,10,10,2,10,10)^′^ and $\boldsymbol {\sigma }^{2}_{\kappa }=(3^{2},1^{2},3^{2},3^{2},1^{2},3^{2},3^{2})'$

*τ*∼Uniform(0,1)

***α***∼Normal(**0**,10^2^**I**)

*p*_*t*_∼Beta(1,1) for *t*≠2017,2018,2019

*p*_*t*_∼Beta(44.04937,13.40566) for *t*=2017,2018,2019

## Appendix 2

**Table 2:** Full version of table 1 from the main text that includes detection probabilities

Parameter	Lower bound	Mean	Upper bound
*β*_0_	16.25	16.36	16.48
*β*_1_	-1.89	-1.77	-1.66
*β*_2_	0.21	0.29	0.35
*β*_3_	0.14	0.22	0.29
*β*_4_	0.14	0.17	0.21
*β*_5_	0.37	0.45	0.55
*β*_6_	-0.37	-0.24	-0.10
*β*_7_	-1.48	-1.34	-1.16
*α*_0_	-1.77	-1.66	-1.55
*α*_1_	2.78	3.16	3.58
*α*_2_	0.12	1.87	6.91
*γ*	0.28	0.29	0.31
*τ*	0.03	0.03	0.03
*θ*_MI_	119.65	147.53	175.46
*θ*_BI_	8.06	9.75	11.40
*θ*_NI_	8.33	9.96	11.63
*θ*_KB_	65.41	98.90	132.59
*θ*_YB_	8.36	10.01	11.69
*θ*_YI_	63.74	96.18	128.84
*θ*_CS_	68.19	98.82	130.55
*κ*_MI_	25.41	28.78	32.20
*κ*_BI_	1.37	2.54	3.80
*κ*_NI_	4.42	9.11	13.90
*κ*_KB_	0.51	0.63	0.78
*κ*_YB_	0.80	2.23	3.79
*κ*_YI_	4.17	8.49	12.40
*κ*_CS_	4.59	9.23	13.91
*p*_1999_	0.74	0.80	0.85
*p*_2000_	0.70	0.75	0.80
*p*_2001_	0.82	0.86	0.89
*p*_2002_	0.86	0.89	0.91
*p*_2003_	0.77	0.79	0.82
*p*_2004_	0.73	0.77	0.81
*p*_2005_	0.53	0.58	0.63
*p*_2006_	0.71	0.75	0.78
*p*_2010_	0.87	0.90	0.92
*p*_2012_	0.54	0.58	0.63
*p*_2017_	0.67	0.77	0.85
*p*_2018_	0.67	0.77	0.85
*p*_2019_	0.67	0.77	0.85

## Data Availability

Data are available from [30, 31], and [33].
